# Antisolvent‐ and Annealing‐Free Deposition for Highly Stable Efficient Perovskite Solar Cells via Modified ZnO

**DOI:** 10.1002/advs.202002860

**Published:** 2021-05-07

**Authors:** Ziyu Wang, Xuejie Zhu, Jiangshan Feng, Chenyu Wang, Cong Zhang, Xiaodong Ren, Shashank Priya, Shengzhong (Frank) Liu, Dong Yang

**Affiliations:** ^1^ Dalian National Laboratory for Clean Energy iChEM Dalian Institute of Chemical Physics Chinese Academy of Sciences 457 Zhongshan Road Dalian 116023 China; ^2^ University of Chinese Academy of Sciences Beijing 100049 China; ^3^ Key Laboratory of Applied Surface and Colloid Chemistry Ministry of Education Shaanxi Engineering Lab for Advanced Energy Technology School of Materials Science and Engineering Shaanxi Normal University Xi'an 710119 China; ^4^ Materials Science and Engineering Pennsylvania State University University Park PA 16802 USA

**Keywords:** chelation, efficiency, perovskite solar cells, stability, ZnO

## Abstract

Even though ZnO is commonly used as the ETL in the perovskite solar cell (PSC), the reactivity of perovskite deposited thereupon limits its performance. Herein, an ethylene diamine tetraacetic acid‐complexed ZnO (E‐ZnO) is successfully developed as a significantly improved electron selective layer (ESLs) in perovskite device. It is found that E‐ZnO exhibits higher electron mobility and better matched energy level with perovskite compared to ZnO. In addition, in order to eliminate the proton transfer reaction at the ZnO/perovskite interface, a high quality perovskite film fabrication process that requires neither annealing nor antisolvent is developed. By taking advantages of both E‐ZnO and the new process, the highest efficiency of 20.39% is obtained for PSCs based on E‐ZnO. Moreover, the efficiency of unencapsulated PSCs with E‐ZnO retains 95% of its initial value exposed in an ambient atmosphere after 3604 h. This work provides a feasible path toward high performance of PSCs, and it is believed that the present work will facilitate transition of perovskite photovoltaics in flexible and tandem devices since the annealing‐ and antisolvent‐free technology.

## Graphical abstract

The highest efficiency of 20.39% is obtained for perovskite solar cells based on modified ZnO without annealing and antisolvent process. The perovskite devices exhibit unprecedented environmental stability owing to efficient decreased organic ligands on ZnO surface.

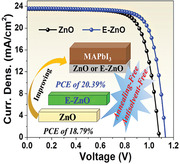

Organic–inorganic hybrid halide perovskites demonstrate admirable optoelectronic properties such as long charge diffusion length, high carrier mobility, large light‐extinction, and tunable bandgap thanks to their excellent electronic band structure, polaronic charge transfer, electron–phonon coherence, and chemical variety.^[^ ^1^, ^2^, ^3^, ^4^, ^5^ ^]^ These outstanding features make the perovskite perfect material for photovoltaic applications. In fact, the power conversion efficiency (PCE) of the perovskite solar cells (PSCs) have been increased from 3.8% in 2009 to as high as 25.2%, making it the highest efficiency among all thin film photovoltaics.^[^ ^6^, ^7^ ^]^ With its simple structure, easy fabrication process, and low cost, the highly efficient PSC has been regarded as a promising next‐generation photovoltaic technology. However, the instability of PSCs due to their sensitivity to moisture and oxygen has seriously limited its commercial application.

Electron selective layers (ESLs) used in PSCs play an important role in device performance such as stability and efficiency. A superior ESL should display good optical transparency in visible spectrum, well‐matched energy level with perovskite material, as well as high electron mobility. TiO_2_ and SnO_2_ have been widely used in PSCs as ESL to give efficiency over 23%.^[^ ^8^, ^9^ ^]^ In high efficient PSCs, the photogenerated carriers should be separated and extracted effectively at the interface between perovskite layer and charge transporting layers. However, TiO_2_ and SnO_2_ show low‐grade electron mobility, which is much lower than the hole mobility of the widely used hole selective layers such as doped poly[bis(4‐phenyl)(2,4,6‐trimethylphenyl)amine and 2,2′,7,7′‐tetrakis[*N*,*N*‐di(4‐methoxyphenyl)amino]‐9,9′‐spirobifluorene (spiro‐OMeTAD),^[^ ^8^, ^10^ ^]^ that would result in charge accumulation and thus degrade the device performance. ZnO has the similar physical properties to TiO_2_ and SnO_2_, but much higher electron mobility and well matched energy level to that of perovskite materials.^[^ ^11^ ^]^ ZnO could be fabricated in many methods such as RF sputtering, atomic layer deposition (ALD), and sol–gel method, etc.^[^ ^12^, ^13^, ^14^, ^15^ ^]^ which make ZnO easy to be crystallized and doped to further improve its properties.

However, the instability of perovskite film deposited on ZnO resulting in poor device stability, and thus makes ZnO underutilized in PSCs.^[^ ^16^ ^]^ Previous reports reveal that the presence of residual hydroxyl group or acetate ligand on ZnO surface during the sol–gel fabrication process could accelerate the decomposition of perovskite especially under annealing process.^[^ ^17^, ^18^ ^]^ These organic groups on ZnO surface lead to the deprotonation of methylammonium cation at perovskite/ZnO interface, leading to the decomposition reaction from perovskite to PbI_2_. Even though the removal of these groups by calcination could result in a more thermal stable perovskite film, the high temperature process lead to enhanced cost and ZnO nanoparticles agglomerated after annealing temperature over 400 °C, resulting in decreased performance.^[^ ^19^ ^]^ To some extent, combustion synthesis, magnetron sputtering, and ALD methods overcome these issues.^[^ ^20^ ^]^ However, these strategies involve long time, complicated process and expensive facilities, which increase the fabrication cost of PSCs.

In order to improve the stability of perovskite deposited on ZnO and simultaneously save the advantages of sol–gel ZnO, two types of modification for ZnO have been investigated previously: doping ZnO or introducing a modified layer on ZnO to improve the surface properties.^[^ ^21^, ^22^ ^]^ Vaynzof et al. doped ZnO with lithium through sol–gel process, and then deposited a self‐assembled monolayer (SAM) of PCBA on ZnO film to modify its surface properties. The efficiency of PSCs was up to 18% with enhanced stability.^[^ ^20^ ^]^ Jang et al. synthesized a polar molecule (named JTCA) and used it to modify ZnO surface through self‐assemble process. The SAM on ZnO improved the charge extraction properties, and the PCE reached 18.82%.^[^ ^23^ ^]^ Ahn et al. doped ZnO with alkali metal by dipping the fresh ZnO film into various alkali‐metal hydroxide solutions (LiOH, NaOH, and KOH). This doped process reduced the surface hydroxyl groups, passivated surface defects, raised the Fermi energy level, and improved electron mobility of ZnO ESLs, resulting in a PCE of 19.90% with remarkable stability.^[^ ^24^ ^]^ Although these approaches improved the performance of PSCs, they also involved more cost and low photocurrent than those of pristine ZnO.

Here, we developed the ethylene diamine tetraacetic acid (EDTA) complexed ZnO (E‐ZnO) as the ESLs in PSCs, because EDTA is a strong chelating agent. The measurements reveal that E‐ZnO has a higher electron mobility, more suitable energy level, and better charge extraction capacity compared to ZnO. More importantly, EDTA efficiently chelates organic ligands on ZnO surface to avoid the decomposition of perovskite. Considering the proton transfer reactions in perovskite/ZnO interface during the annealing process, we explored an annealing‐ and antisolvent‐free method to fabricate methylammonium‐based perovskite (MAPbI_3_) films. As a result, the PSCs based on E‐ZnO exhibits a PCE of 20.39%, which is the highest efficiency in the PSCs with ZnO, with excellent environmental stability.

X‐ray photoelectron spectroscopy (XPS) measurements were performed to study elemental distribution of ZnO chelated with EDTA. **Figure** **1**a shows the XPS survey spectra of ZnO and E‐ZnO films deposited on glass/ITO substrates. It is obviously that E‐ZnO shows N1s peak located at around 400 eV,^[^ ^25^ ^]^ which is contributed to the introduction of EDTA. Meanwhile, the Zn2p peaks of E‐ZnO are shifted to high binding energy by ≈0.19 eV compared to ZnO (Figure S1, Supporting Information), indicating indeed reaction between ZnO and EDTA. Fourier transform infrared (FTIR) spectra were conducted to investigate the chemical interaction between ZnO and EDTA, as showed in Figure 1b. The peak around 1693 cm^−1^ in EDTA belongs to C=O stretching vibration, while it disappears in E‐ZnO and appears an extra new peak at 1595 cm^−1^, which is attributed to the stretching vibration of O—C—O.^[^ ^26^ ^]^ This transformation from C=O stretching vibration to O—C—O stretching vibration demonstrates that oxygen‐containing groups such as hydroxyl group or acetate ligand on ZnO surface are significantly decreased, which effectively improves the stability of PSCs, because the reduced organic groups suppress the deprotonation of methylammonium cation between perovskite and ZnO.

**Figure 1:**
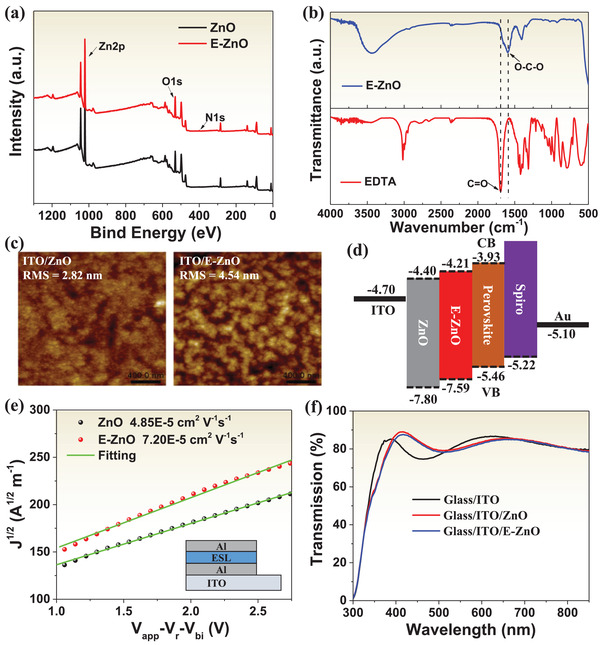
a) XPS of ZnO and E‐ZnO deposited on ITO substrates. b) FTIR spectra of EDTA and E‐ZnO. c) AFM images of ZnO and E‐ZnO films. d) Schematic illustration of energy level of ZnO and E‐ZnO relative to those of the perovskite layer. e) Electron mobility of ZnO and E‐ZnO using the SCLC model. The structure of electron‐only device is showed in the inset. f) Transmission spectra of ZnO and E‐ZnO films on ITO substrate.

Figure 1c shows the atomic force microscopy (AFM) images of ZnO and E‐ZnO. It can be seen that the root‐mean‐square roughness of ZnO and E‐ZnO are 2.82 and 4.54 nm, respectively, revealing that both ZnO and E‐ZnO film exhibit relatively smooth surface, which is beneficial to the performance of PSCs. The relatively bigger spot size of E‐ZnO may be caused by the chelation between EDTA and ZnO. Their correspond scanning electron microscope (SEM) images of ZnO and E‐ZnO show consistent results, as shown in Figure S2 of the Supporting Information.

The Fermi level (E_F_) of ZnO and E‐ZnO were measured by ultraviolet photoelectron spectrometer (UPS). The UPS data were shown in Figure S3 of the Supporting Information. The E_F_ can be calculated using equation E_F_ = E_cut_ – 21.22 eV, where E_cut_ is the cut‐off binding energy, and 21.22 eV is emission energy of Helium irradiation. The E_cut_ of ZnO is 17.24 eV, while that of E‐ZnO shifts to 17.38 eV. The E_F_ of ZnO and E‐ZnO are thereby −3.98 and −3.84 eV, respectively. The Fermi edge (E_edge_) of ZnO and E‐ZnO are 3.82 and 3.75 eV (Figure S3, Supporting Information), respectively. The valence band (E_VB_) can be calculated by E_VB_ = E_F_ − E_edge_. Therefore, the E_VB_ of ZnO and E‐ZnO are −7.80 and −7.59 eV, respectively. According to the Tauc plot from absorption spectra (Figure S4, Supporting Information), the band gap (E_g_) of ZnO (3.40 eV) and E‐ZnO (3.38 eV) can be obtained. The corresponding conduction band (E_CB_) of ZnO and E‐ZnO can be calculated as −4.40 and −4.21 eV according to equation of E_CB_ = E_g_ + E_VB_. Thus, the energy level diagram of perovskite devices is shown in Figure 1d. The energy level of ITO, perovskite, spiro‐OMeTAD, and Au are obtained from literatures.^[^ ^25^, ^27^, ^28^, ^29^ ^]^ The conduction band of E‐ZnO (−4.21 eV) is much closer to the conduction band of perovskite (−3.93 eV) than that of ZnO (−4.40 eV), which is beneficial to reducing energy loss in perovskite devices.^[^ ^30^ ^]^ The electron mobility of ZnO and E‐ZnO were measured using the space charge‐limited current (SCLC) method,^[^ ^31^ ^]^ as shown in Figure 1e. It is apparent that E‐ZnO displays higher electron mobility of 7.20 × 10^−5^ cm^2^ V^−1^ s^−1^ than that of ZnO (4.85 × 10^−5^ cm^2^ V^−1^ s^−1^). The calculated details can be seen in the Supporting Information. The high electron mobility promotes electron extraction from perovskite into ESLs, which consist with photoluminescence (PL) spectra, as will discuss below. Figure 1f shows the optical transmission spectra of ZnO and E‐ZnO films coated on glass/ITO substrates. Both films exhibit high average transmittance from 400 to 800 nm, which guarantees enough photons to be transmitted into perovskite absorber.

Above results reveal that E‐ZnO is very suitable application in perovskite devices as the ESLs. Therefore, we deposited MAPbI_3_ on ZnO and E‐ZnO surface using solution method. It should be noted that in order to eliminate the proton transfer reaction at the ZnO/perovskite interface, we developed a high quality perovskite film fabrication process that requires neither annealing nor antisolvent. Specifically, we prepared MAPbI_3_ acetonitrile solution using presynthesized MAPbI_3_ single crystal, and then this solution was dynamically applied onto the substrate during spin‐coating. The perovskite film was immediately formed neither antisolvent nor annealing. The formation process can be seen in Video S1 of the Supporting Information, and the details of the fabrication procedures can be found in the Experimental Section. The crystallinity of the MAPbI_3_ films is investigated by X‐ray diffraction (XRD), as shown in Figure S5 of the Supporting Information. MAPbI_3_ deposited on ZnO and E‐ZnO substrates show sharp characteristic peaks, demonstrating very high crystallinity, which is a prerequisite for high performance for PSCs.

**Figure** **2**a,b shows the morphology of perovskite films deposited on ZnO and E‐ZnO substrates. It is apparent that uniform pinhole‐free films with full coverage have been obtained. These films show smaller grain size compared with previous work focused on annealing and antisolvent process,^[^ ^32^, ^33^ ^]^ that may be caused by the fast crystallization due to the volatility of acetonitrile solvent. The grain size distribution diagram shows an average grain size of about 130 nm when the perovskite deposited on ZnO, while it slightly increases to around 190 nm for the E‐ZnO substrate, as shown in Figure 2c. The grain size of perovskite film is relevance with the surface properties of the substrate. Therefore, the water contact angles of ZnO and E‐ZnO substrates were measured. The contact angle of E‐ZnO slightly reduces to 42.0° from 49.7° for ZnO surface, as shown in Figure S6 of the Supporting Information, which assists to form perovskite film due to polarity of perovskite precursor solution. The wettability of E‐ZnO substrate provides the low surface tension,^[^ ^34^ ^]^ resulting in formation of relative larger perovskite grain during the growth of the networked structure,^[^ ^35^ ^]^ as seen in SEM images. The large grain size facilitates to improvement of efficiency and stability for PSCs.

**Figure 2:**
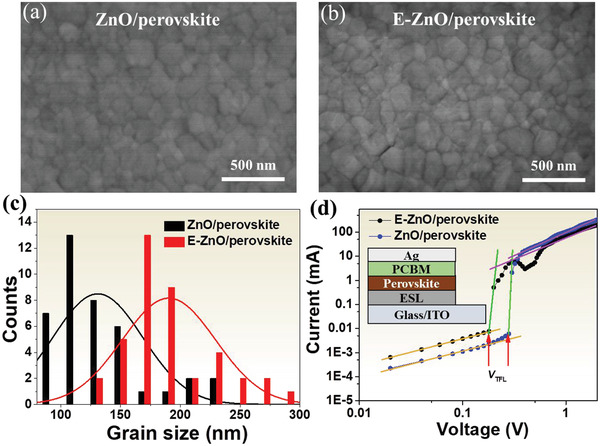
SEM images of perovskite films coated on a) ZnO and b) E‐ZnO. c) The grain size distribution diagram. d) Dark *I*–*V* curves of the single carrier devices with the *V*_TFL_ kink points. The inset shows the structure of the single carrier device.

The single carrier devices were fabricated to evaluate the trap density within perovskite deposited on ZnO and E‐ZnO substrates, and inset in Figure 2d gives the structure of single carrier device. Figure 2d shows the dark current–voltage (*I*–*V*) curves of single carrier devices. The linear correlation (yellow line) reveals an ohmic‐type response at low bias voltage. When the bias voltage is above the kink point, which defines as the trap‐filled limit voltage (*V*_TFL_), the current nonlinearly increase (green line), indicating that the traps are completely filled. The trap density within perovskite coated on E‐ZnO decreases to 2.29 × 10^15^ from 3.44 × 10^15^ cm^−3^ for ZnO. The detailed calculation can be found in the Supporting Information. The lower trap density of perovskite deposited on E‐ZnO would efficiently suppress the charge recombination, leading to high performance of PSCs.

The performance of solar cells is always the gold standard for assessment of ESLs. Therefore perovskite devices using ZnO and E‐ZnO as the ESLs were fabricated, and the structure is glass/ITO/ZnO or E‐ZnO/MAPbI_3_/spiro‐OMeTAD. MAPbI_3_ was fabricated by annealing‐ and antisolvent‐free approach, which efficiently avoids the proton transfer reactions between ZnO and MAPbI_3_ during perovskite growth, significant benefiting to perovskite stability. Figure S7 of the Supporting Information gives the cross‐sectional SEM images of complete devices based on ZnO and E‐ZnO. The thickness of MAPbI_3_ and spiro‐OMeTAD are 554 and 167 nm. The thickness of ZnO (43 nm) and E‐ZnO (45 nm) is almost same, which can eliminate the influence of device performance by the thickness.

**Figure** **3**a shows the *J*–*V* curves of PSCs using different ESLs, and inset summarizes the parameters, including the open‐circuit voltage (*V*_oc_), short‐circuit current density (*J*_sc_), fill factor (FF), and PCE. The device based on ZnO displays a PCE of 18.79% with *J*_sc_ of 23.52 mA cm^−2^, *V*_oc_ of 1.083 V, and FF of 73.8%. While the device based on E‐ZnO gives a PCE of 20.39% with *J*_sc_ of 23.59 mA cm^−2^, *V*_oc_ of 1.1134 V, and FF of 76.2%. The high efficiency is mainly contributed to large *V*_oc_ and FF. The higher *V*_oc_ is attributed to the closer energy between E‐ZnO and perovskite (Figure 1d), which can reduced energy loss during electron transfer from perovskite into E‐ZnO. The larger FF is ascribed to high electron mobility of E‐ZnO and decreased trap density of perovskite deposited on E‐ZnO, which could reduce the charge recombination. We also measured their *J*–*V* responses scanned at reverse and forward directions, as shown in Figure S8 of the Supporting Information. Although each device shows very high PCE scanned at reverse direction, it provides very low value scanned at forward direction. The hysteresis maybe originate from ion migration in perovskite and relative small perovskite grains using annealing‐free method.^[^ ^36^, ^37^, ^38^, ^39^ ^]^ Figure 3b shows the incident‐photon‐to‐charge conversion efficiency (IPCE) and the integrated photocurrent of the PSCs based on E‐ZnO. The integrated photocurrent is 22.22 mA cm^−2^, close to the *J*–*V* result. Figure 3c shows the histogram distribution of PCE for 27 individual perovskite devices based on ZnO and E‐ZnO, with the statistics summarized in Tables S1 and S2 of the Supporting Information. Both types of PSCs exhibit good reproducibility with a small standard deviation.

**Figure 3:**
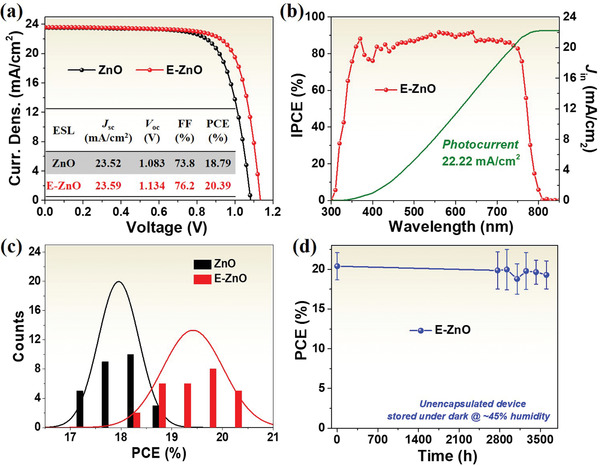
a) *J*–*V* characteristics and b) IPCE spectra of PSCs with ZnO and E‐ZnO. c) Histogram distribution of PCE for PSCs based on ZnO and E‐ZnO ESLs. d) Environmental stability of unencapsulated perovskite device with E‐ZnO stored in the dark under an ambient atmosphere for 3604 h.

Figure 3d shows the long‐term stability of PSC with E‐ZnO, and the parameters are listed in Table S3 of the Supporting Information. Compared to perovskite device based on ZnO reported by other groups,^[^ ^20^, ^40^, ^41^ ^]^ our device exhibits amazed long term environmental stability. The efficiency of device retains about 95% of its initial value when stored in the air (a humidity of about 45%) under the dark for 3604 h. The excellent stability is due to reduced organic ligand on ZnO surface by EDTA (Figure 1b), efficiently suppressing the deprotonation of methylammonium cation at perovskite and ZnO interface. The long‐term stability of device with ZnO fabricated by our method was measured under the same conditions, as shown in Figure S9 and Table S4 of the Supporting Information. The device also exhibits superior environmental stability, which is caused by the annealing‐free strategy that effectively avoids the protons transfer at ZnO and MAPbI_3_ interface during perovskite growth. Meanwhile, we measured the thermal stability of devices based on E‐ZnO and ZnO, as shown in Figure S10 of the Supporting Information. The thermal stability of devices with E‐ZnO and ZnO is operated at 85 °C hot plate in glove box for 800 min. Device based on ZnO keeps 76% of its initial PCE after 800 min, while the device with E‐ZnO retains 93% of its initial value under the same conditions, indicating that the perovskite device with E‐ZnO displays the good thermal stability.

The charge transfer dynamics in the perovskite devices were studied by photoluminescence (PL) and time‐resolved PL (TRPL). **Figure** **4**a shows the steady‐state PL spectra of the perovskite films deposited on different substrates. Compared to other samples, ITO/E‐ZnO/perovskite displays the weakest PL intensity, indicating that E‐ZnO possesses the highest electron extraction capacity. Figure 4b shows the normalized TRPL for perovskite films deposited on different substrates. The fitting parameters are summarized in Table S5 of the Supporting Information, and fitting details can be seen in the Supporting Information. In general, the slow decay lifetime (*τ*_1_) is attributed to radiative recombination of free charge carriers due to traps in the bulk, and the fast decay lifetime (*τ*_2_) originates from the quenching of charge carriers at the interface.^[^ ^42^ ^]^ Compared to ZnO/perovskite, *τ*_2_ significantly decreases to 24.98 from 37.83 ns when the perovskite deposited on E‐ZnO. The reduced fast decay lifetime indicates more efficient charge transfer at E‐ZnO/perovskite interface. This is one reason why perovskite devices based on E‐ZnO exhibit high efficiency.

**Figure 4:**
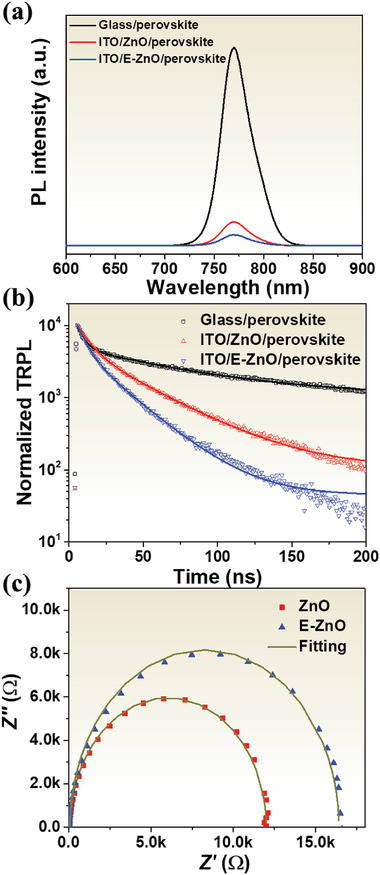
a) Steady‐state PL and b) TRPL spectra of perovskite films deposited on different substrates. c) EIS of PSCs with ZnO and E‐ZnO ESLs.

The electrical impedance spectroscopy (EIS) was performed to investigate the resistance of charge transfer and recombination in perovskite devices. Figure 4c shows the Nyquist plots of the devices based on ZnO and E‐ZnO measured at their *V*_oc_ under dark condition. The equivalent circuit of EIS is shown in Figure S11 of the Supporting Information, and fitting data are summarized in Table S6 of the Supporting Information. It is clear that the charge transfer resistance (*R*_tr_) decreases to 165 Ω from 428 Ω for ZnO when using E‐ZnO. While the charge recombination resistance (*R*_rec_) increases to 15 158 Ω for E‐ZnO from 11 316 Ω for ZnO. The small *R*_tr_ and large *R*_rec_ are beneficial to charge transport in perovskite devices. This is another reason why perovskite devices with E‐ZnO have good performance.

In conclusion, we developed a novel E‐ZnO ESL that shows the high electron mobility, the ideal matched energy level with perovskite materials, and the reduced residual organic ligands on ZnO surface. In order to eliminate the proton transfer reaction at the ZnO/perovskite interface, we developed a high quality perovskite film fabrication process that requires neither annealing nor antisolvent. By taking advantages of both E‐ZnO and the new process, we attained unprecedented PCE of 20.39% based on E‐ZnO, 9% higher than the device using regular ZnO. The long‐term environmental stability of PSCs is significantly improved using the E‐ZnO with 95% of its initial efficiency remained after 3604 h of exposure in air ambient. This is the best stability of PSCs using ZnO ETL to our knowledge. Meanwhile, the devices show the good thermal stability. This breakthrough will promise to be applied in flexible and tandem device since annealing‐free and superior stability of perovskite.

## Experimental Section

##### Materials

Methylamine solution (33 wt% in absolute ethanol), PbI_2_ (99%), and hydroiodic acid (HI) (57 wt% in water, *γ*‐butyrolactone (GBL, 99%) were purchased from Sigma‐Aldrich and used as received without further purification. EDTA, Zn(CH_3_COO)_2_·2H_2_O, 2‐methoxyethanol and ethanolamine were purchased from Alfa Aesar and without any purification.

##### Synthesis of MAI

MAI was synthesized and purified according to a previous reported procedure.^[^ ^31^ ^]^ Generally, CH_3_NH_2_ and HI were mixed with the molar ration of 1.2:1 in a round‐bottom flask and stirred at 0 °C for 3.5 h. The precipitate was obtained by using rotary evaporator at 60 °C to remove the solvents. The product was redissolved in absolute ethanol and precipitated by adding enough diethyl ether. Those steps were repeated twice to obtain the purificated MAI. Finally, the MAI powder was dried at 60 °C in a vacuum oven overnight.

##### Synthesis of MAPbI_3_ Single Crystals

MAPbI_3_ single crystals were synthesized according to the previous reported procedure.^[^ ^43^ ^]^ PbI_2_ and MAI were mixed with molar ratio of 1:2 and dissolved in GBL under vigorous stirring to form a 1.2 m solution. The solution was filtered and placed into an oven at 100 °C for several days to obtain the black crystals.

##### Synthesis of MAPbI_3_ Acetonitrile Solution

MAPbI_3_ acetonitrile solution was prepared according to the previous reported procedure.^[^ ^44^ ^]^ 0.50 g MAPbI_3_ crystals were placed in an opened vail and then transferred into a bigger bottle containing methylamine solution. After several hours, the black crystals were reacted with the diffused CH_3_NH_2_ vapor and resulting in yellow liquid perovskite intermediate. Finally, the yellow intermediate was dissolved in 1 mL acetonitrile to form the 0.8 m MAPbI_3_ acetonitrile solution.

##### ESLs Fabrication

ZnO and E‐ZnO precursor solutions were produced according to the previous reports.^[^ ^26^, ^45^ ^]^ The 1 g zinc acetate dihydrate (Zn(CH_3_COO)_2_·2H_2_O) and 0.28 g ethanolamine were dissolved in 10 mL 2‐methoxyethanol under vigorous stirring for 12 h. EDTA was dissolved into the same mixed solvent with ZnO precursor solution. To get the E‐ZnO precursor solution, the two solutions were mixed together with EDTA concentration of 1 mg mL^−1^ and stirred at 85 °C. The ZnO and E‐ZnO layers were fabricated by spin‐coating at 2000 rpm for 60 s, and then transferred onto a hot plate at 200 °C for 1 h to obtain the ZnO and E‐ZnO films.

##### Device Fabrication

The precursor solution was dynamically dropped onto the cleaned glass/ITO substrates during spinning at 4000 rpm. And then the 0.8 m MAPbI_3_ acetonitrile solution was spin‐coated on ZnO and E‐ZnO substrates. After 60 s, a black perovskite film was fabricated without annealing process. Then, the spiro‐OMeTAD solution (90 mg spiro‐OMeTAD, 36 µL of 4‐tert butylpyridine, and 22 µL of lithium bis(tri‐fluoromethylsulfonyl) imide of 520 mg mL^−1^ in acetonitrile were dissolved in 1 mL chlorobenzene) was coated on perovskite film at 5000 rpm for 30 s with accelerated speed of 3000 rpm s^−1^. The samples were retained in a desiccator for 12 h to oxide the spiro‐OMeTAD. Finally, 100 nm thick Au was deposited on spiro‐OMeTAD using thermal evaporation. The cell area of 0.09 cm^2^ was defined by a metal mask.

##### Characterization

*J*–*V* curves of the PSCs were measured by Keithley 2400 source at reverse and forward directions under ambient condition, and the scan rate was 0.1 V s^−1^, the scan step was 0.02 V. The illumination intensity was calibrated to 100 mW cm^−2^ using an NREL‐traceable KG5 filtered silicon cell. The device area is 0.09 cm^2^, which defined by a metal mask. IPCE was carried out the QE‐R system (QE‐R3011, Enlitech, UCMC & US), and the light source is a 150 W halogen tungsten lamp. AFM images were achieved by a Bruker Multimode 8 in tapping mode. Steady‐state PL (excitation at 510 nm) and TRPL were tested by Rudower Chaussee 29 (PicoQuant GmbH, Geman). XRD patterns were obtain by a Rigaku Smart Lab (X‐ray Source: Cu K*α*; *λ* = 1.54 Å).

## Conflict of Interest

The authors declare no conflict of interest.

## Supporting information

Supporting Information

Supplemental Video 1
