# Supplementary material for: Antisolvent‐ and Annealing‐Free Deposition for Highly Stable Efficient Perovskite Solar Cells via Modified ZnO
Source: Adv Sci (Weinh). 2021 May 7;8(13):2002860. doi: 10.1002/advs.202002860 (PMC8261502; doi:10.1002/advs.202002860)
Supplement: Supplementary file 1 — Supporting Information [file ADVS-8-2002860-s001.pdf]

© 2020 Wiley-VCH GmbH

ADVANCED SCIENCE Open Access

Supporting Information

for *Adv. Sci.*, DOI: 10.1002/advs.202002860

Antisolvent- and Annealing-Free Deposition for Highly Stable Efficient Perovskite Solar Cells via Modified ZnO

Ziyu Wang, Xuejie Zhu, Jiangshan Feng, Chenyu Wang, Cong Zhang, Xiaodong Ren, Shashank Priya, Shengzhong (Frank) Liu,* and Dong Yang**

Supporting Information

Antisolvent- and Annealing-Free Deposition for Highly Stable Efficient Perovskite Solar Cells via Modified ZnO

Ziyu Wang, Xuejie Zhu, Jiangshan Feng, Chenyu Wang, Cong Zhang, Xiaodong Ren, Shashank Priya, Shengzhong (Frank) Liu,* and Dong Yang**

Z. Wang, Prof. S. Liu
Dalian National Laboratory for Clean Energy, iChEM, Dalian Institute of Chemical Physics, Chinese Academy of Sciences, 457 Zhongshan Road, Dalian 116023, China
E-Mail: szliu@dicp.ac.cn
X. Zhu, Dr. J. Feng, C. Wang, C. Zhang, Dr. X. Ren, Prof. S. Liu
Key Laboratory of Applied Surface and Colloid Chemistry, Ministry of Education; Shaanxi Engineering Lab for Advanced Energy Technology, School of Materials Science and Engineering, Shaanxi Normal University, Xi'an 710119, China
Prof. Priya, Dr. D. Yang
Materials Science and Engineering, Pennsylvania State University, University Park, Pennsylvania 16802, United States
E-mail: sup103@psu.edu; dzy82@psu.edu
Z. Wang
University of Chinese Academy of Sciences, Beijing 100049, China

Calcalated electron mobility of ZnO and E-ZnO

The elecron-only devices with structure of glass/ITO/Al/ESL/Al were fabricated to calculate the electron mobility of ZnO and E-ZnO. The dark *J-V* curves of the electron-only devices based on ZnO and E-ZnO were shown in Figure 1e. The electron mobility is calculated by the SCLC according to following eqation:[1]

$$J = \frac{9}{8}\varepsilon_0\varepsilon_r\mu_e\frac{(V_{app} - V_r - V_{bi})^2}{L^3}$$

where J is the current density, ε_0 is the vacuum permittivity, ε_r is the dielectric permittivity of the ESL, L is the thickness of ESL, V_{app} is the applied voltage of the device, V_r is the voltage drop due to constant resistance and series resistance across the electrodes,[2] V_{bi} is the built-in voltage due to the different WF of the two electrodes (this value is 0 in this work because the two electrodes used same materials),[3] and μ_e is the electron mobility. The electron mobility of ZnO and E-ZnO are 4.83×10^{-5} cm^2 V^{-1} s^{-1} and 7.20×10^{-5} cm^2 V^{-1} s^{-1}, respectively.

Caculated trap density within perovskite films

The structure of single carrier device is glass/ITO/ESL/$MAPbI_3$/PCBM/Ag, and the dark *I-V* curves are shown in Figure 2d. The trap-filled limit voltage (V_{TFL}) can be obtain from the drak *I-V* plots. The trap density (N_t) can be calculated using following equation:[4]

$$N_t = 2\frac{\varepsilon_0\varepsilon_r V_{TFL}}{eL^2}$$

where ε_0 is the vacuum permittivity, ε_r of 28.8 is the relative dielectric constant of $MAPbI_3$,[5] e is the elementary charge, L is the thickness of the $MAPbI_3$ film. The trap density of $MAPbI_3$ deposited on ZnO and E-ZnO are 3.44×10^{15} cm^{-3} and 2.29×10^{15} cm^{-3}, respectively.

TRPL fitting

TRPL spectra are fitted by a biexponential equation:

$$f(t) = A_1 \exp\left(-\frac{t}{\tau_1}\right) + A_2 \exp\left(-\frac{t}{\tau_2}\right) + B$$

where τ_1 and τ_2 are slow and fast decay lifetime, respectively, A_1 and A_2 are their corresponding decay amplitude, and B is a constant.

References

[1] D. Yang, L. Zhou, W. Yu, J. Zhang, C. Li, *Adv. Energy Mater.* **2014**, *4*, 1400591.

[2] S. Park, J. H. Heo, C. H. Cheon, H. Kim, S. H. Im, H. J. Son, *J. Mater. Chem. A* **2015**, *3*, 24215.

[3] G. G. Malliaras, J. R. Salem, P. J. Brock, C. Scott, *Phys. Rev. B* **1998**, *58*, 13411.

[4] R. H. Bube, *J. Appl. Phys.* **1962**, *33*, 1733.

[5] A. Poglitsch, D. Weber, *J. Chem. Phys.* **1987**, *87*, 6373.

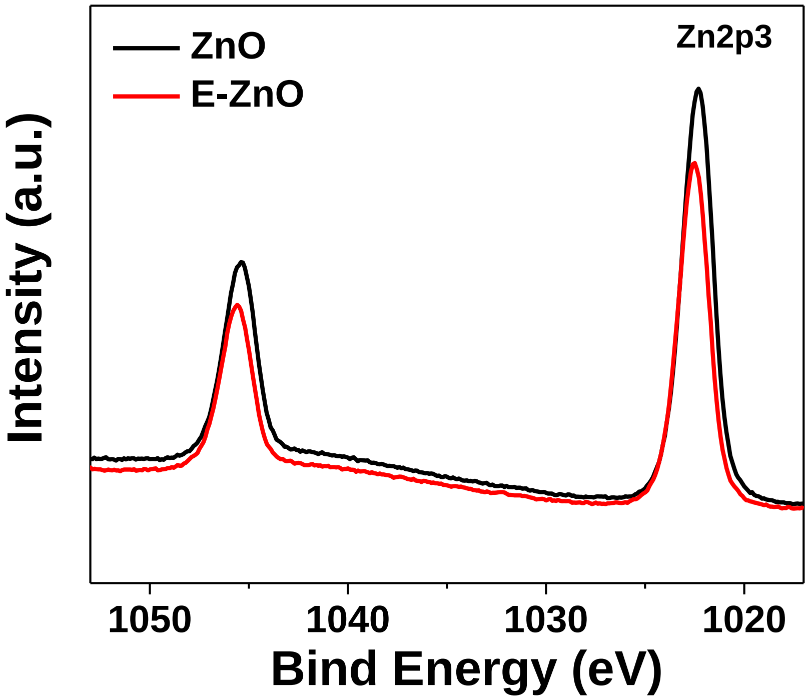

Figure S1. XPS analysis of Zn2p of ZnO and E-ZnO films.

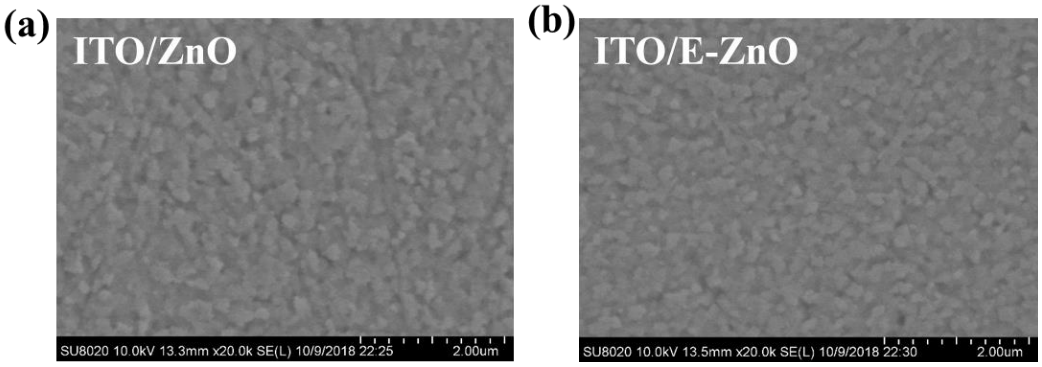

Figure S2. SEM image of **(a)** ZnO and **(b)** E-ZnO deposited on ITO substrates.

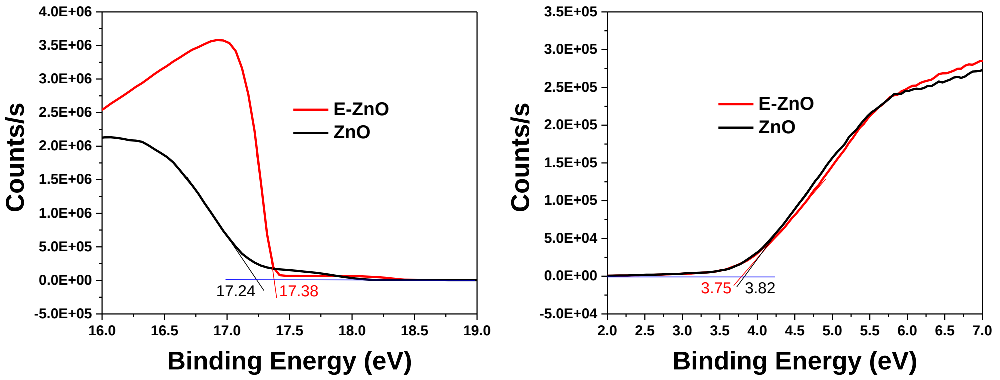

Figure S3. UPS results of ZnO and E-ZnO.

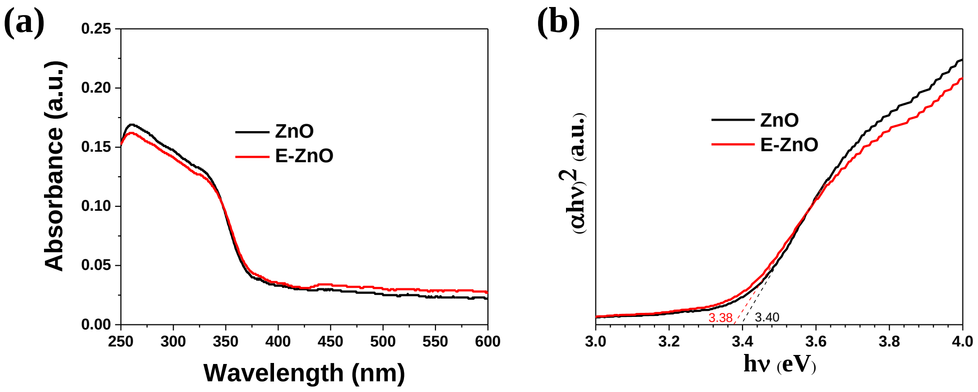

Figure S4. (a) UV-vis absorption spectra and (b) tauc plots of ZnO and E-ZnO.

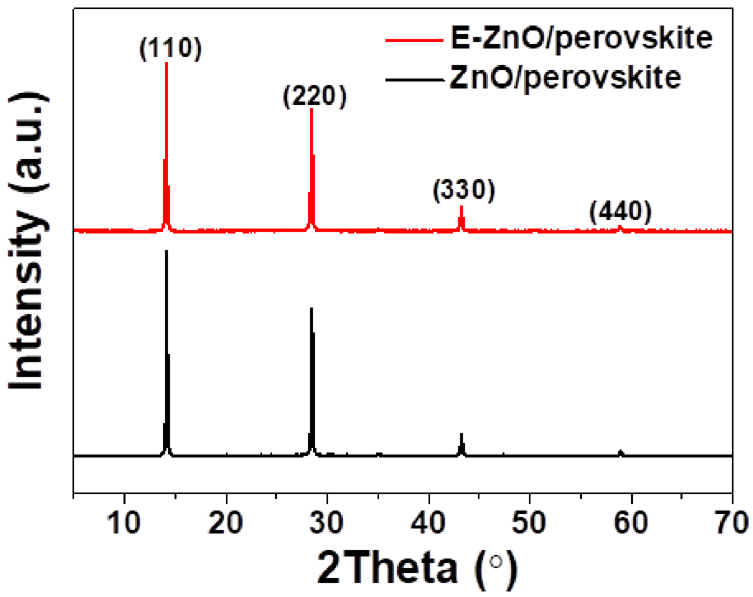

Figure S5. XRD patterns of perovskite films deposited on ZnO and E-ZnO.

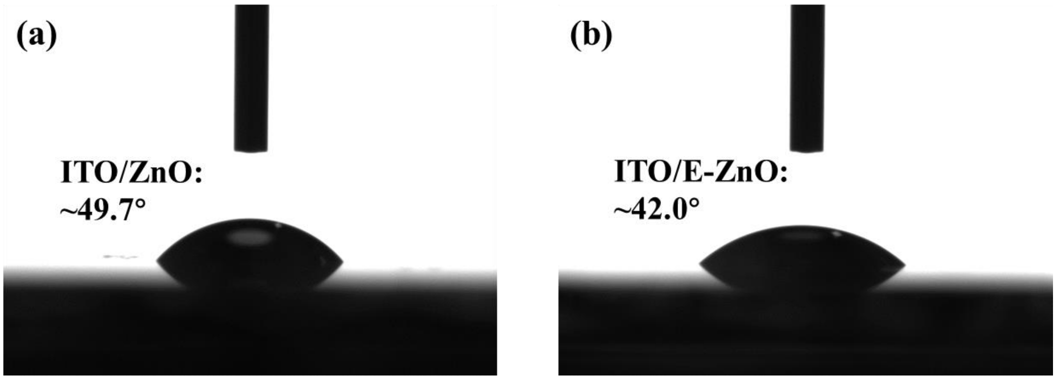

Figure S6. Water contact angles of **(a)** ITO/ZnO and **(b)** ITO/E-ZnO.

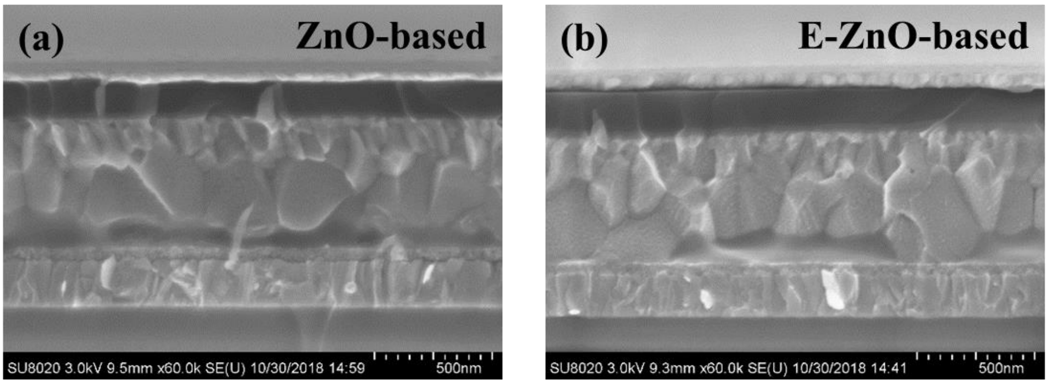

Figure S7. Cross-sectional SEM images of complete perovskite devices based on **(a)** ZnO and **(b)** E-ZnO ESLs.

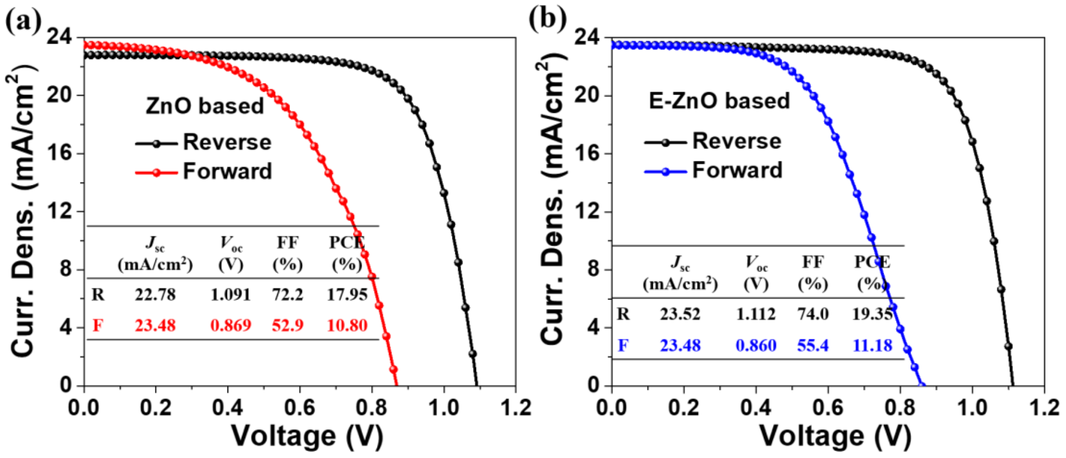

Figure S8. *J-V* curves of PSCs based on **(a)** ZnO and **(b)** E-ZnO scanned at reverse and forward directions. Inset is their *J-V* parameters.

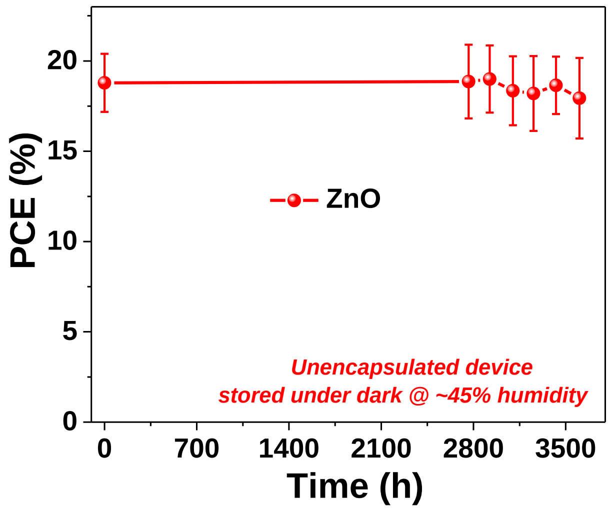

Figure S9. Environmental stability of unencapsulated perovskite device with ZnO stored in the dark under an ambient atmosphere for 3604 hours.

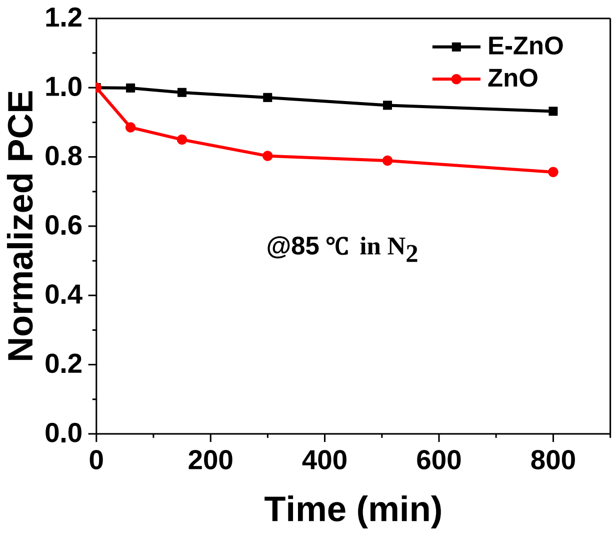

Figure S10. Thermal stability of ZnO and E-ZnO based devices. The devices kept on the hot plate at 85 ℃ in glove box.

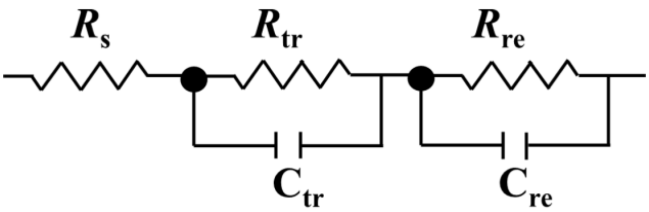

Figure S11. The equivalent circuit model for EIS.

Table S1. Parameters of PSCs based on ZnO.

	J_{sc} (mA/cm^2)	V_{oc} (V)	FF (%)	PCE (%)
**1**	23.67	1.075	71.8	18.27
**2**	23.36	1.085	71.3	18.06
**3**	23.60	1.052	70.9	17.60
**4**	23.58	1.036	71.5	17.47
**5**	23.55	1.046	72.6	17.90
**6**	23.54	1.084	72.8	18.56
**7**	23.52	1.083	73.8	18.79
**8**	23.45	1.084	73.6	18.73
**9**	23.68	1.053	72.9	18.18
**10**	23.59	1.028	71.8	17.41
**11**	23.39	1.051	71.7	17.63
**12**	23.38	1.061	72.2	17.91
**13**	23.51	1.044	72.9	17.89
**14**	23.52	1.041	72.6	17.76
**15**	23.65	1.056	72.5	18.09
**16**	23.65	1.052	72.3	17.98
**17**	23.43	1.064	73.2	18.24
**18**	23.43	1.060	72.9	18.11
**19**	23.31	1.051	73.6	18.05
**20**	23.73	1.115	68.8	18.21
**21**	23.66	1.093	70.0	18.11
**22**	23.40	1.098	69.7	17.90
**23**	23.58	1.057	68.8	17.16
**24**	23.71	1.040	70.2	17.31
**25**	23.57	1.057	70.1	17.47
**26**	23.68	1.068	71.2	18.01
**27**	23.67	1.065	71.1	17.92
**Average**	23.55 ± 0.12	1.063 ± 0.021	71.7 ± 1.4%	17.95 ± 0.40

Table S2. Parameters of PSCs based on E-ZnO.

	J_{sc} (mA/cm^2)	V_{oc} (V)	FF (%)	PCE (%)
**1**	23.56	1.096	73.3	18.92
**2**	23.59	1.088	72.8	18.67
**3**	23.40	1.124	73.9	19.44
**4**	23.40	1.134	74.8	19.83
**5**	23.16	1.129	75.3	19.70
**6**	23.16	1.126	75.1	19.58
**7**	23.48	1.125	74.8	19.75
**8**	23.93	1.096	73.2	19.21
**9**	23.83	1.119	74.9	19.96
**10**	23.72	1.128	75.4	20.19
**11**	23.59	1.134	76.2	20.39
**12**	23.73	1.107	73.5	19.33
**13**	23.73	1.105	73.3	19.21
**14**	23.52	1.112	74.0	19.35
**15**	23.01	1.146	76.1	20.12
**16**	22.98	1.131	72.6	18.88
**17**	23.71	1.135	75.0	20.18
**18**	23.78	1.132	75.1	20.23
**19**	23.88	1.119	73.9	19.74
**20**	23.87	1.117	73.6	19.62
**21**	23.91	1.111	74.3	19.75
**22**	23.41	1.094	71.4	18.28
**23**	23.57	1.095	72.7	18.76
**24**	23.50	1.095	73.3	18.86
**25**	23.59	1.082	72.8	18.60
**26**	23.70	1.101	74.4	19.43
**27**	23.33	1.093	71.8	18.31
**Average**	23.56 ± 0.26	1.114 ± 0.018	74.0 ± 1.2	19.42 ± 0.60

Table S3. The stability parameters of the PSC based on E-ZnO stored in the air under dark.

Stored time (hours)	J_{sc} (mA/cm^2)	V_{oc} (V)	FF (%)	PCE (%)
0	23.59	1.134	76.2	20.39
2763	23.35	1.144	74.4	19.86
2923	23.11	1.146	75.4	19.97
3099	23.14	1.118	72.6	18.79
3256	23.29	1.131	75.1	19.78
3426	22.97	1.130	75.7	19.65
3604	22.50	1.131	75.8	19.30

Table S4. The stability parameters of the PSC based on ZnO stored in the air under dark.

Stored time (hours)	J_{sc} (mA/cm^2)	V_{oc} (V)	FF (%)	PCE (%)
0	23.52	1.083	73.8	18.79
2763	23.35	1.115	72.4	18.86
2923	23.44	1.112	72.9	19.00
3099	23.57	1.064	73.2	18.35
3256	23.29	1.069	73.1	18.20
3426	23.49	1.091	72.8	18.65
3604	22.86	1.089	72.1	17.94

Table S5. Fitting parameters for TRPL spectra of perovskite films coated on different substrates.

**Sample**	**τ_{ave} (ns)**	**τ_1 (ns)**	**Amplitude of τ_1 (%)**	**τ_2 (ns)**	**Amplitude of τ_2 (%)**
Glass/perovskite	82.11	5.39	17.13	97.97	82.87
ZnO/perovskite	28.82	7.71	67.68	37.83	32.32
E-ZnO/perovskite	17.79	6.30	71.26	24.98	28.74

Table S6. Fitting parameters of EIS for ZnO and E-ZnO.

ETL	R_s (Ω)	R_{tr} (Ω)	CPE_{tr} (F)	R_{rec} (Ω)	CPE_{rec} (F)
ZnO	33	428	3.56×10^{-9}	11316	5.54×10^{-9}
E-ZnO	26	165	1.78×10^{-9}	15185	3.88×10^{-9}
